# Comfort Evaluation of Slow-Recovery Ejection Seat Cushions Based on Sitting Pressure Distribution

**DOI:** 10.3389/fbioe.2021.759442

**Published:** 2021-11-30

**Authors:** Jiayi Bao, Qianxiang Zhou, Xingwei Wang, Chao Yin

**Affiliations:** ^1^ School of Biological Science and Medical Engineering, Beihang University, Beijing, China; ^2^ Beijing Advanced Innovation Centre for Biomedical Engineering, Beihang University, Beijing, China; ^3^ Air Force Medical Center, Beijing, China

**Keywords:** comfort evaluation, sitting pressure distribution, ejection seat cushion, pilots healthcare, slow-recovery materials

## Abstract

Sitting discomfort not only affects the health of pilots carrying out long-endurance missions but also affects operational performance. The experimental objects included four ejection seat cushions: N1 was a fast-recovery foam as the comparison group, and the experimental groups were slow-recovery foams with different indentation force deflection (IFD), named N2 (hard), N3 (mid), and N4 (soft). The sitting comfort of 20 participants was tested on the four cushions by using subjective rating and sitting pressure distribution analysis. The results showed that compared with fast-recovery cushion N3 and N4 slow-recovery cushions have lower contact pressure and more uniform pressure distribution. Slow-recovery cushions that were too soft or too hard would reduce the comfort. No matter from the subjective rating or the analysis of the contact pressure data, the N3 cushion with a thickness of 3 cm and 65% IFD of 280 N had the highest comfort. In addition, the seat pressure distribution (SPD%) has a significant correlation with the subjective rating (*p* = 0.019, R = −0.98), which is more suitable for evaluating the comfort of the cushions. However, the slow-recovery cushions would show a decrease in support after a period of sitting, while the fast-recovery cushion could always maintain constant support.

## Introduction

The ejection seat system is designed to optimize the safety of fighter pilots during ejection rather than their comfort when sitting on the seat (Ojetola et al., 2011). However, in repeated training and long-endurance missions, attention has been focused on the comfort of the seat. According to the response of Chinese fighter pilots, the lack of comfortable seats makes them suffer from severe muscle pain and fatigue. This discomfort affects their mission performance. Pint et al. (2002) reported that the current US Air Force pilots had physiological problems such as buttock and leg pain, numbness, tingling, and general fatigue during extended missions due to the discomfort of ejection seat cushions. Excessive local pressure at the person–seat interface will produce soft tissue deformation, which will result in discomfort by restricted blood and nutrient flow (Kumar et al., 1994). Pilots who have been sitting in this uneven pressure distribution for a long time will have pressure sores and venous thrombosis, which will seriously affect their health (Wu et al., 1998). In addition, chronic back pain, as one of the problems caused by sitting for a long time, is a major physical health disorder and may lead to mental health disorder, which also has an impact on work performance (Akkarakittichoke and Janwantanakul, 2017). Pellettiere and Cheng (2004) reported that the problem of chronic back pain in military pilots has become more serious. In another report, he proposed that the cushion of the ejection seat is usually the only component that can be improved to mitigate these effects (Pellettiere et al., 2006). Improving the comfort of the ejection seat cushion is necessary for the pilot's health and flight performance.

The comfort of the seat is determined by its design factors such as material, structure, size, tilt angle, etc. (Dénes et al., 2020). The material, shape, and stiffness of the seat cushion are further characteristics that determine the comfort of sitting (Kapica and Grbac, 1998). Brienza et al. (1996), Go and Lee (2017), and Li et al. (2020) proposed that if the shape of the seat cushion is more suitable for the human body, it can better disperse the pressure and improve comfort. Kumar et al. (2019) and Mohanty and Mahapatra (2014) demonstrated that the sitting comfort increases as the thickness of the seat cushion increases, and comfort will no longer change until the thickness exceeds 6–8 cm. Lee et al. (2016), Zemp et al. (2016), and Moon et al. (2020) found that the material and structure of the seat cushion have a significant influence on sitting comfort. And the influence is shown in many aspects such as stiffness, supporting force, and air permeability. However, the improvement of the comfort of the ejection rescue system must be based on ensuring safety. Due to many restrictions on the size of the aircraft cabin, the special structure of the seat, and the mechanical requirements, very limited change can be made. Some scholars have verified the safety of different ejection seat cushions (Adams and Lankarani, 2003; Cheng and Pellenttiere, 2004; Pellettiere and Cheng, 2004; Perry, 2007). Beheshti and Lankarani (2006) proved that the thickening of the seat cushion will increase the lumbar load during ejection, which will pose a threat to the pilot's life safety. The maximum allowable thickness of ejection seat cushions on Chinese combat aircraft is 3 cm, and changing the seat cushion configuration to optimize comfort is very limited. Hearon and Brinkley (1986), Cheng and Pellettiere (2005), Perry (2007), and Wang and Dal Nevo (2018) demonstrated that the slow-recovery material seat cushion has better safety than the common foam seat cushion when an ejection occurs.

The slow-recovery material (memory foam) was developed by the National Aeronautics and Space Administration (NASA) in 1962 in order to improve the protection of the seat by absorbing the huge impact of the spacecraft during take-off and return to earth (Luo et al., 2017). The special viscosity characteristic of slow rebound gives it a strong impact absorption ability. In addition, the open cell structure of the slow-recovery material has fluidity, which can slowly adapt to the entire contact surface, thus evenly dispersing the pressure (Mills et al., 2003). It is widely used in various fields of comfort optimization, in seat cushions (Roslim et al., 2018), insoles (Wang et al., 2017), pillows (Jeon et al., 2014), earplugs (Casali and Park, 1990), etc. and has a good performance. The properties of slow-recovery materials vary greatly, and among these properties, stiffness has the most direct effect on cushion comfort. Since flexible foam usually has obvious viscoelasticity, its stiffness is expressed by indentation force deflection (IFD). Therefore, in this experiment, three kinds of slow-recovery seat cushions with different IFD and an ordinary fast-recovery cushion used in combat aircraft as the comparison were selected as the research objects of comfort.

The study of cushion comfort can be divided into dynamic and static. Dynamic comfort refers to the cushion's ability to mitigate the discomfort caused by the vibration of the vehicle (Basri and J, 2014; Beard and Griffin, 2014; Ciloglu et al., 2015). The present study focuses on static ride comfort. The research methods of static comfort include subjective surveys and objective data analysis. In terms of the subjective survey, some scholars put comfort and discomfort on the same dimension (0 is extremely uncomfortable, 5 or 10 is extremely comfortable). Li et al. (2020) used this to rate the overall comfort of the seat cushion; more researchers have used the measure to rate the comfort in different areas of the body (Ebe and Griffin, 2001; Stubbs et al., 2005; Cascioli et al., 2011), while Kamijo et al. (1982) and Zhang et al. (1996) proposed that comfort and discomfort are independent concepts with different influencing factors. Zhang et al. (1996) found that comfort is related to people's feelings of relaxation and pleasure, and discomfort is related to feelings of fatigue, numbness, and soreness caused by physical stimulation. Based on these descriptions, he proposed a multidimensional comfort scale. The former survey method is more suitable for the overall design of the seat. For the design of the cushion, people's feelings are mainly concentrated in the bottom and legs, so this survey method has limitations. Using the latter survey method can evaluate the comfort of the cushion more comprehensively from multiple dimensions. Objective measurements included sitting posture analysis (Mohanty and Mahapatra, 2014), electromyography (Harrison et al., 1999), blood oxygen (Parakkat et al., 2006), and sitting pressure distribution. Pressure distribution seems to be the objective measure most clearly related to the subjective survey (De Looze et al., 2003). Measuring the sitting pressure distribution can provide designers with fast and easy to quantify data. These data can indicate which areas have an impact on the comfort of the seat in the early stages of the design process (Gyi et al., 1998). It is the most commonly used method in the comfort design of the seat. Therefore, in the present study, the comfort multidimensional survey and the sitting pressure distribution analysis were selected to evaluate the comfort of the ejection seat cushion.

Among the many parameters in the sitting pressure distribution, the average pressure, peak pressure, contact area, and pressure gradient are more used in the comfort evaluation of the cushion (Jackson et al., 2009; Smardzewski et al., 2014; Guo et al., 2016; Yongxiang et al., 2019). Zemp et al. (2016) and Liu et al. (2018) removed the repeated sitting pressure distribution parameters through correlation analysis and also screened out these four parameters as evaluation indicators. Pressure gradient refers to the uniformity of pressure distribution, which has an important relationship with sitting comfort (Milivojevich et al., 2000). Ahmadian et al. (2002) proposed a new parameter, Seat Pressure Distribution (SPD%), to calculate the uniformity of pressure distribution. The calculation formula is as follows:

$$SPD\%=\frac{\sum_{i=1}^{n}{\left(p_{i}-p_{m}\right)}^{2}}{4np_{m}^{2}}\times 100$$

*P*_*i*_ is the pressure on the *i*th unit, *p*_*m*_ is the average pressure, and *n* is the total number of pressure points with a non-zero value. SPD% can be used to calculate the pressure distribution in static and dynamic environments. It characterizes the uniformity of the overall body pressure distribution on the cushion: the smaller, the more uniform. And it has a good effect on the comfort research of the cushions (Smardzewski et al., 2014; Hu et al., 2018; Campos and Xi, 2020).

For research on the comfort of ejection seats, Ojetola et al. (2011) and Qiu et al. (2012) studied the impact of seat inclination on comfort. Pint et al. (2002) and Cheng and Pellettiere (2005) analyzed the comfort of current cushions in the US military and found that slow-recovery cushions generally have better performance, but their comfort is also related to material property. Parakkat et al. (2006) proposed a new dynamic buffering cushion to improve comfort and proved that different genders have different preferences for cushions. Pellettiere and Gallagher (2007) studied the time dependence of the subjective comfort evaluation of the seat cushion and proved that the subject's preference for the seat cushion will no longer change after 6 h.

Applying slow-recovery materials to the comfort optimization of ejection seat cushions seems to be a good choice. However, the material properties of slow-recovery foams vary greatly. Which kind of foam is more suitable for the ejection seat cushions? The purpose of the present study is to verify, as a cushion for ejection seat, 1) whether slow-recovery foams have better comfort than ordinary fast-recovery foams and 2) how the slow-recovery ejection cushions with different IFD affect sitting comfort.

## Methods

### Participants

Participants were recruited based on the height (157.9–181.9 cm) and weight (48.5–94.5 kg) range of Chinese fighter pilots. Twenty healthy postgraduates (10 males and 10 females) from Beihang University aged between 22 and 28 years, with 60.25 (±9.37) kg mean weight, 169.55 (±7.54) cm mean height, and 20.85 (±2.20) kg/m^2^ mean body mass index (BMI) participated in the experiment. Musculoskeletal disorders such as scoliosis and bulging intervertebral discs were excluded after examination by CT scan. Meanwhile, all participants were told not to have strenuous physical activities and to have adequate rest the day before the test. On the day of the test, they were asked to wear soft and light sports pants. The experimental protocol was approved by the Ethics Committee of Beihang University, all methods were carried out by relevant guidelines and regulations, and written informed consent was obtained from the participants before the experiment.

### Experimental Objects and Apparatus

The sitting test was carried out on the fourth-generation ejection seat of Chinese combat aircraft, and the experimental cushions were also manufactured according to the shape and size of this ejection seat. The experimental objects included four kinds of cushions, named from N1 to N4: as the comparison group, N1 was an ordinary fast-recovery cushion that has been used on ejection seats in China; N2 to N4 were slow-recovery cushions with different IFD. Some mechanical parameters of the cushion material are shown in Table 1, from the manufacturer Liming Research Institute of Chemical Industry (Luoyang, China).

**TABLE 1:** Mechanical parameters of cushion materials.

		N1	N2	N3	N4	Test standard
Density (kg/m³)		72	74	70	69	GB/T 6343–2009
Tensile strength (Kpa)		164	249	168	150	GB/T 6344–2008
Elongation at break (%)		96	108	137	150	GB/T 6344–2008
Tear strength (N/m)		286	554	404	225	GB/T 10808–2006
IFD (N)	**25%**	290	220	136	87	GB/T 10807–2006
	**50%**	458	308	157	124	
	**65%**	860	460	280	174	
	**75%**	1670	930	560	261	
	**85%**	6,730	3,940	2,230	579	

Bold represents the IFD from 25% to 85%.

**FIGURE 1:**
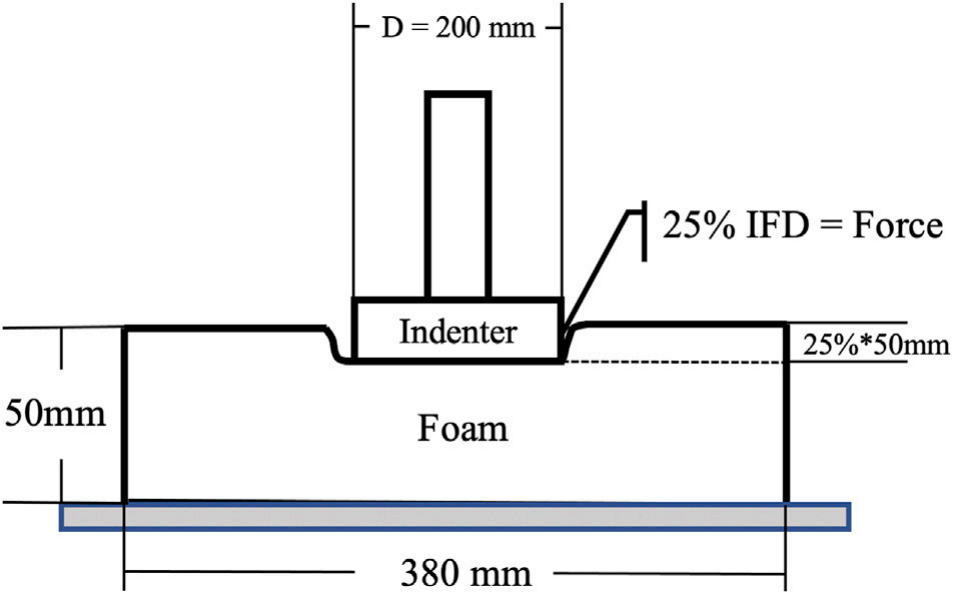
The schematic of IFD test.

**FIGURE 2:**
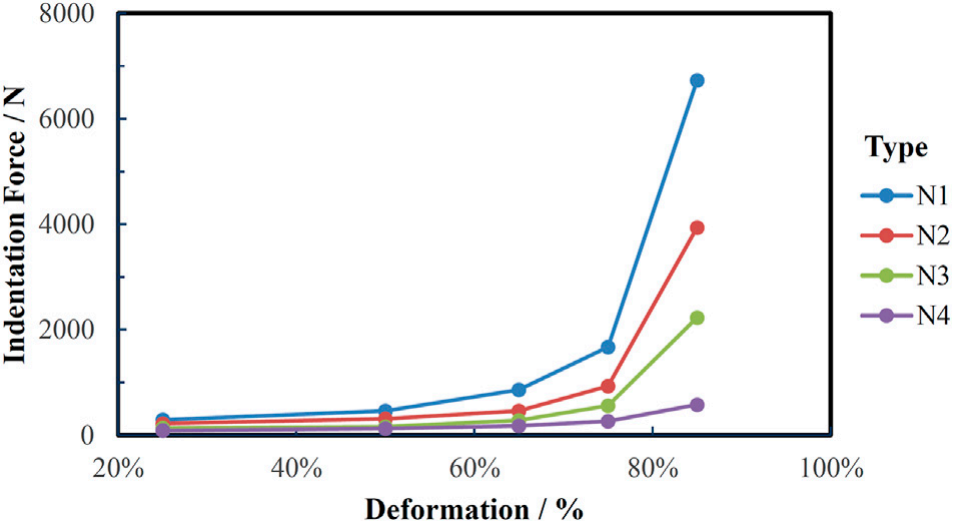
The indentation force of the experimental cushions under different deformation.

According to the test standard GB/T 10807-2006, IFD comes from uniaxial compression test. As shown in Figure 1, the diameter of the circular indenter is 200 mm, the accuracy is ±1 N, and the movement speed is 100 mm/min. The size of the test foam sample is 380 × 380 × 50 (±2) mm. After compressing to the specified depth, keep the fixed displacement for 30 s and then read the force value corresponding to the IFD. As described in Figure 2, N2 had the largest IFD, N3 was medium, and N4 was the softest. In addition, The Tekscan CONFORMat (Tekscan, Boston, United States), which has 1,024 sensors in a 32 × 32 matrix, was used to measure the pressure distribution.

### Experimental Procedures

The experiment was carried out in a room with a constant temperature of 25°C and a relative humidity of 40%. Each participant tested the comfort of the four cushions separately. The rank of the cushion test was random to avoid the influence of the order. The experiment is divided into two parts: subjective rating and sitting pressure distribution measurement. Before the experiment, the experiment process and the subjective survey content were explained to the participants to ensure that they fully understand the purpose of the experiment and the rule of rating.

**FIGURE 3:**
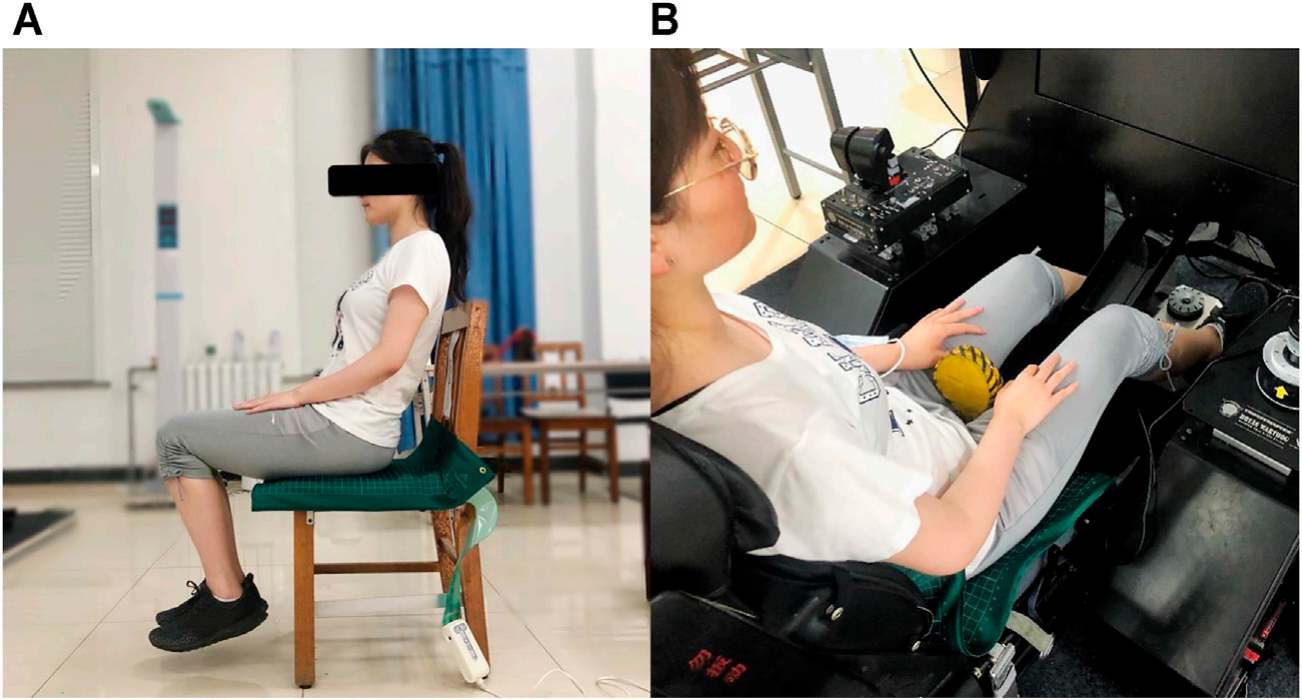
**(A)** The sitting posture of participants to calibrate. **(B)** The sitting posture of participants to collect the pressure.

The height, weight, and BMI of participants without shoes were measured before the sitting test. Because the pressure measurement system tested is the pressure ratio between the sensors, calibration by the weight of each subject was necessary to get real pressure data first. As shown in Figure 3A, the pressure mat was placed on a hard and flat surface. The participant sat gently on it, with both feet off the ground, without any support from the body, and made the entire weight of the body fall on the mat. Then the weight of the subject was input into the CONFORMAT Research 7.20 (Chinese, PRC) software, the automatic sensitivity adjustment for calibration was selected, and it was completed after 60 s.

At the beginning of the experiment, the cushion to be tested was placed on the ejection seat. The participants sat down slowly in the driving posture, relaxed, and leaned back naturally, with their feet on the rudder. The horizontal distance of the seat was adjusted so that the rudder was in a comfortable position, the feet were free to operate, and the thighs were in contact with the front of the seat cushion without a feeling of pressure. According to the guidance of the subjective scale content, the participants carefully experienced the comfort of the cushion. After 15 min of riding, the scale was scored. Then, the participant stood up, and the pressure sensing mat was laid on the cushion. Participants sit upright and gently in the above sitting posture, with their hands naturally resting on their thighs, as shown in Figure 3B. Collected were 150 frames of pressure data after the subject had sat on the cushion for approximately 5 min to allow for material settling. The sampling rate is 8 Hz, and the data were saved in video format.

Participants completed the evaluation of one cushion and rested for 5 min before the next test. After all tests were over, participants were asked to freely experience all cushions and carefully correct the subjective comfort rating to avoid scoring deviation caused by no comparison.

### Subjective Rating Design and Data Processing

Subjective comfort survey is derived from Zhang et al. (1996) and Liu et al. (2018). Zhang gave 18 effective descriptors in terms of comfort and 20 in terms of discomfort. The cushions we evaluated have the same shape, so the descriptors about the appearance such as “luxurious” and “plush” have been removed. All cushions tested are made of soft foams, and descriptors such as “hurting” and “smarting” were removed. Due to the short test time and easy tasks, descriptors such as “fatigue” and “sleeping” were removed. Liu turned these descriptors into short sentences for the participants to easily understand and added a description of the overall comfort, making the scale more applicable. Combining the two research, we finally selected 7 comfortable and 10 uncomfortable descriptors. The rating scores ranged from 1 to 9, meaning from “not at all” to “extremely.”

This survey describes comfort from multiple dimensions. The average calculation cannot reflect the hierarchy between descriptors, which may bias the evaluation results. Therefore, we used the analytic hierarchy process (AHP) method to calculate the priority scales of descriptors. AHP is a measurement theory that obtains a priority scale through pairwise comparison (Saaty, 2008). Comfort as positive scores and discomfort as negative scores were calculated separately. The final selected subjective scale descriptors and corresponding priority scales are indicated in Table 2. For each descriptor, the average score of all participants was multiplied by the priority scales, and then all the items were added to get the subjective rating of the cushion.

**TABLE 2:** Subjective evaluation descriptors and corresponding priority scales.

Descriptors		Priority scales
Comfort	I feel relaxed	0.04
	I feel spirits soared	0.04
	I feel restful	0.04
	I feel softer	0.08
	I feel supported enough	0.08
	I feel refreshed	0.08
	I feel comfortable	0.15
Discomfort	I have sore muscles	−0.04
	I have heavy legs	−0.04
	I feel stiff	−0.04
	I feel tired	−0.04
	I have swollen ankles	−0.04
	I feel numb	−0.04
	I feel the circulation to legs cut off	−0.04
	I feel cramped	−0.04
	I feel restless	−0.04
	I feel uncomfortable	−0.13

The rating scores ranged from 1 to 9, meaning from “not at all” to “extremely.”

### Pressure Distribution Data Processing

Firstly, the data films were preprocessed in CONFORMAT Research: the unstable frames were removed to eliminate the error caused by the slight movement of the subjects at the beginning and the end of the measurement; the useless data caused by the distortion of the edge of the pressure sensing mat were cleared by delimiting the sampling area. Average pressure (Average P), maximum pressure (Max P), and contact area (Contact A) were obtained from the software. Then, the preprocessed movies were exported as a data table including approximately 130 frames; each frame contained a 32 * 32 matrix, which recorded the pressure from each sensor. SPD% was calculated by this data table in the Matlab2019 (vR 2019a, MathWorks Inc., United States) software.

### Statistics Analysis

The results are reported as the mean ± standard deviation (SD). First, all indicators were tested for normality, and then data were analyzed with a repeated-measures analysis of variance (ANOVA). The means were compared by Duncan's test at a probability of 95%. Pearson's two-tailed correlation analysis was performed on the subjective rating and the sitting pressure distribution parameters.

## Result

After normality verification, all indicators in this experiment conformed to the normal distribution. As described in Table 3, through the ANOVA, all parameters had significant differences for the four types of cushions except Contact A. For convenient comparison, we used the N1 value of the control group as the standard and normalized the data.

**TABLE 3:** The comparison of the mean values between the four cushions, using N1 as the standard, and the significance of each parameter to different cushions (*n* = 20).

Parameters	Cushion Type				F	Sig
	N1	N2	N3	N4		
Max P	1.00	1.12	0.83	0.84	F (2.038, 38.730) = 20.477	<0.001
Average P	1.00	1.04	0.91	0.86	F (1.998, 37.968) = 45.015	<0.001
Contact A	1.00	0.98	1.03	1.03	F (1.889, 35.884) = 1.929	0.162
SPD%	1.00	1.11	0.88	0.98	F (3, 57) = 4.992	0.007
Rating	1.00	0.86	1.08	1.01	F (2.667, 50.666) = 6.974	0.001
Sup R	1.00	0.94	0.84	0.77	F (3, 57) = 8.208	<0.001

### Subjective Rating Analysis

**FIGURE 4:**
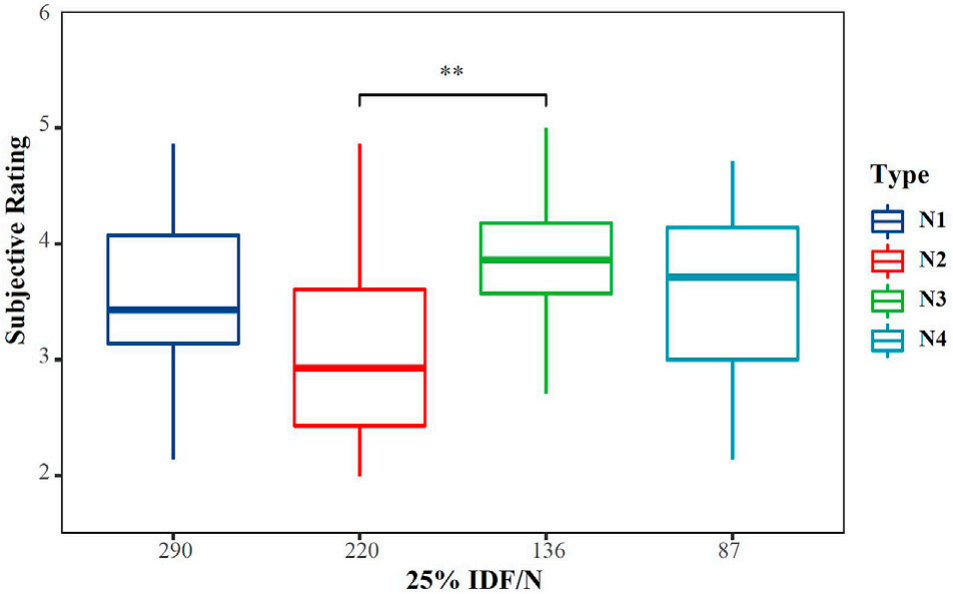
The subjective rating is significantly different for the four types of cushions (*p* = 0.001) (*p < 0.05; **p < 0.01).

**FIGURE 5:**
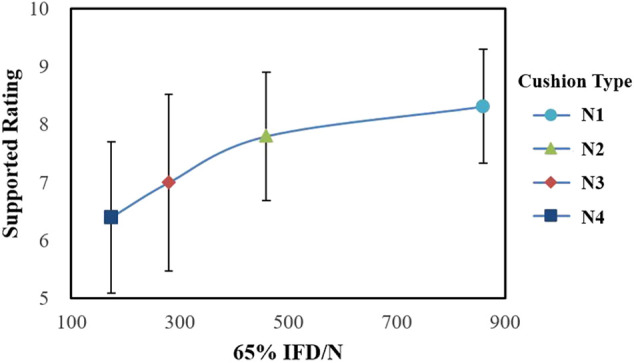
Sup R increases with the increase in 65% IFD.

As indicated in Table 3 and Figure 4, the subjective rating (Rating) is significantly different for the four types of cushions (*p* = 0.001), and the N3 cushion has the highest comfort score. The results of the pairwise comparison N2 and N3 are significantly different (*p* < 0.001), but the *p*-values between the slow-recovery cushions and the fast-recovery cushion both exceed 0.05. We collected participants' suggestions on cushions. Eight participants mentioned that after riding on the N4 cushion for a while, it would become sunken and stuffy, while the support of the N1 cushion was relatively stable. Therefore, we paid special attention to the “I feel supported enough” descriptor (Sup R) and found that 75% of the participants scored fast-recovery cushion greater than or equal to slow-recovery cushions. As shown in Figure 5, there is an obvious difference in 65% IFD, while Sup R increases with the increase in IFD. Compared with N1, N3 has significantly lower Sup R (*p* = 0.005), and the Sup R of N4 is also lower than N1 (*p* = 0.008).

### Pressure Distribution Data Analysis

**FIGURE 6:**
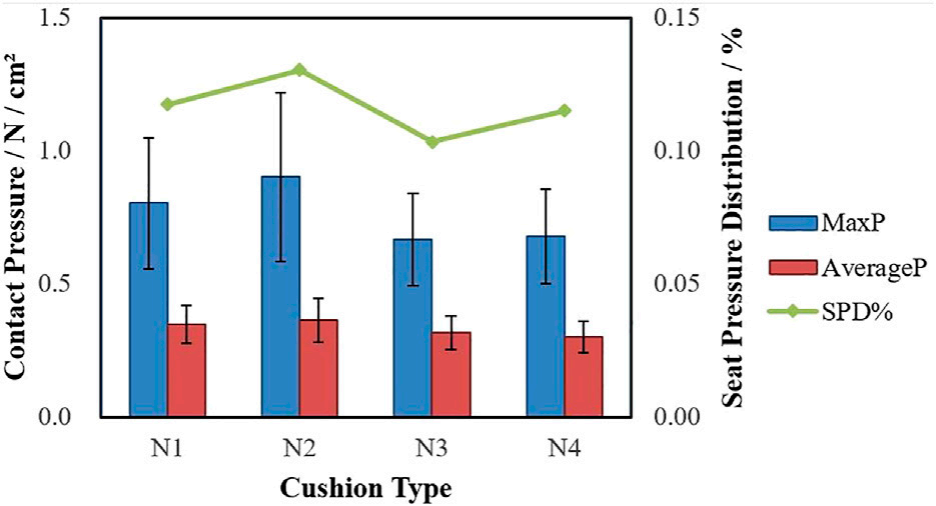
The mean contact pressure ±SD for 20 participants in the four types of cushions.

**FIGURE 7:**
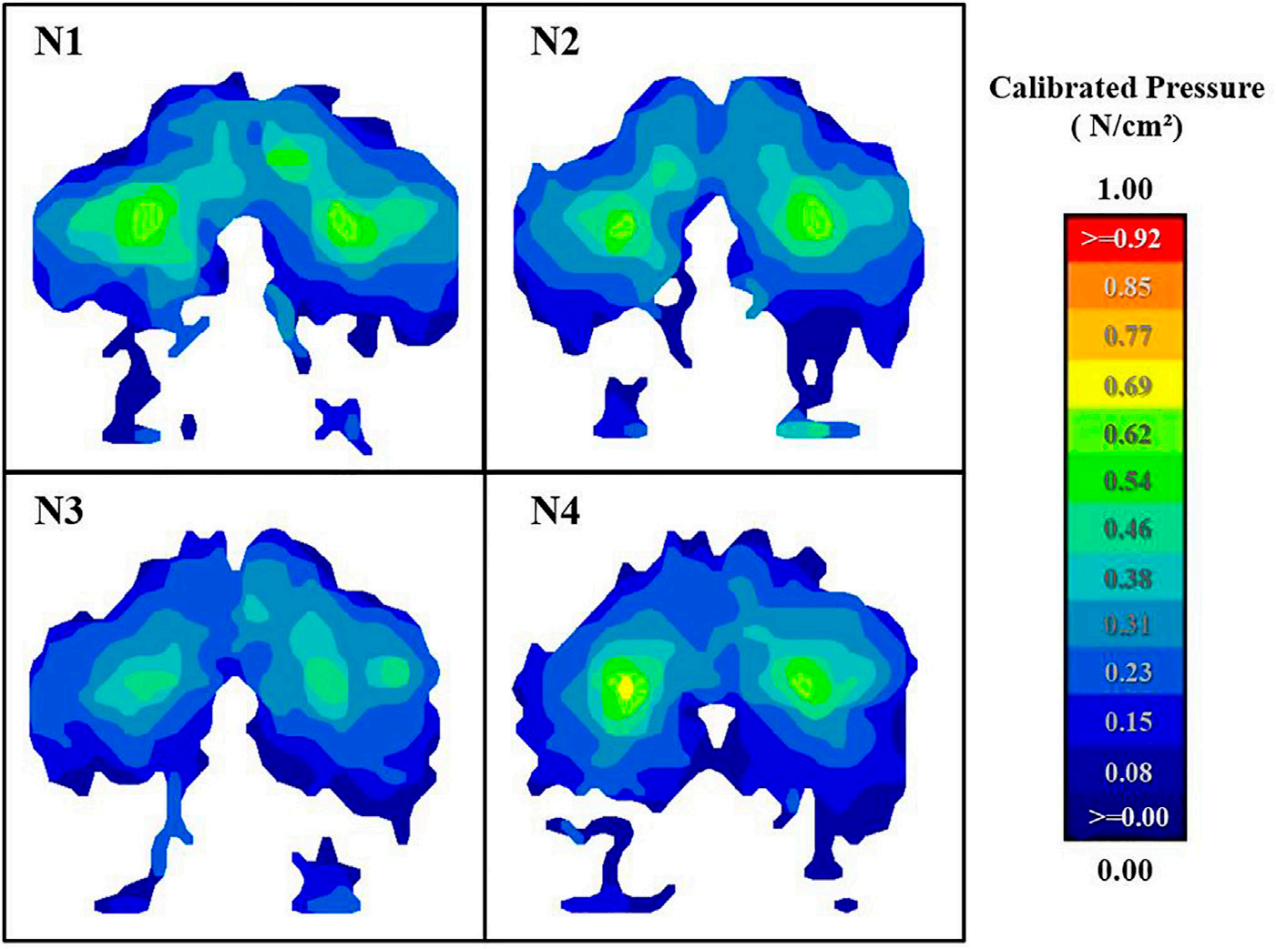
The average pressure distribution of one participant with 60.12 kg sitting on the four types of cushions.

As shown in Figure 6 and Table 3, the contact pressure (*p* < 0.001) and SPD% (*p* = 0.004) have significant differences among the four cushions. The lowest value of Max P occurred at N3. Compared with fast-recovery cushion N1, the Max P of N3 is significantly lower (*p* = 0.001), and N4 is also lower than N1 (*p* = 0.005), while between slow-recovery cushions, the value of N2 is significantly higher than N3 and N4 (*p* < 0.001), the difference between N3 and N4 is not significant. The lowest value of Average P occurred at N4. Compared with fast-recovery cushions, N3 and N4 are significantly lower (*p* < 0.001); the values between slow-recovery cushions are significantly different (*p* < 0.05). The lowest value of SPD% occurred at N3. The contrast between slow-recovery and fast-recovery cushions is not significant, while N2 is significantly higher than N3 (*p* = 0.003). In addition, the pressure nephogram can show the pressure distribution more intuitively. Figure 7 was the pressure nephogram of a participant with 60.12 kg weight sitting on the four types of cushions. The total contact pressures evaluated as integral of the measured contact pressures were respectively N1: 114.62 N/cm^2^, N2: 106.91 N/cm^2^, N3: 95.05 N/cm^2^ and N4: 101.26 N/cm^2^. N3 has the smallest contact pressure and the most uniform pressure distribution.

### Relationship Between Subjective Rating and Pressure Parameters

**FIGURE 8:**
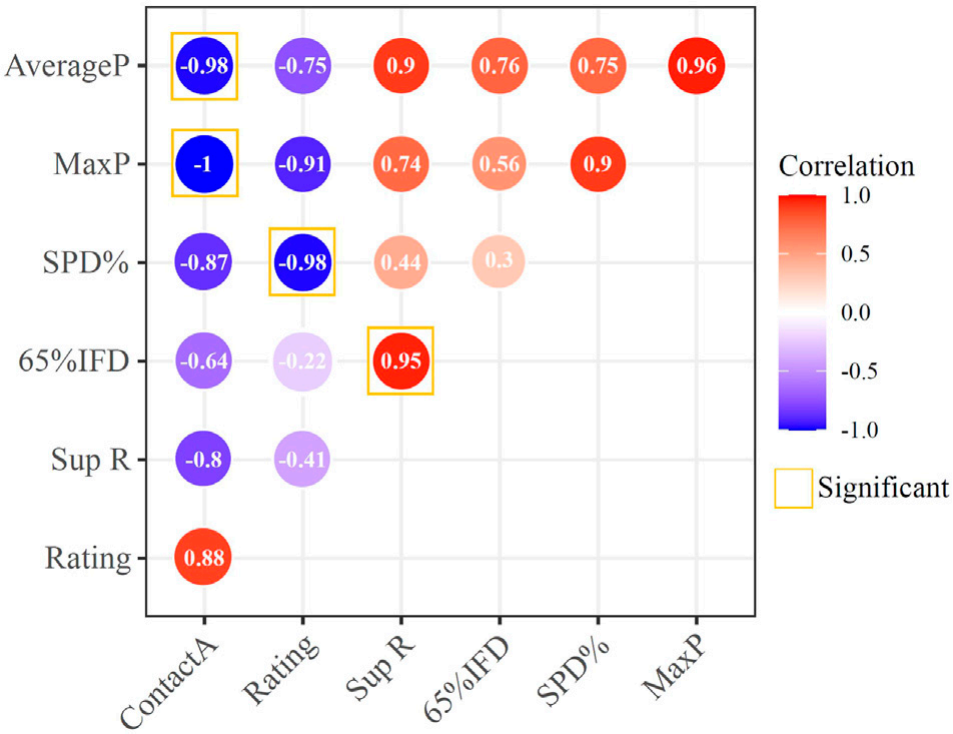
Correlation analysis between subjective rating and pressure parameters.

The correlation analysis between subjective rating and pressure distribution parameters are described in Figure 8: There is a significant negative correlation between Rating and SPD% (*p* = 0.019, R = −0.981), and the correlation between other sitting pressure parameters and Rating are not significant. There is a correlation between Sup R and 65% IFD (*p* = 0.048, R = 0.952). In addition, Contact A and Average P (*p* = 0.04, R = −0.980) and Max P (*p* = 0.001, R = −0.999) also have significant correlations.

## Discussion

Three slow-recovery cushions with different IFD and a fast-recovery cushion as a comparison group were tested using subjective survey and sitting pressure distribution to evaluate comfort characteristics. Subjective rating, maximum pressure, average pressure, contact area, and SPD% were analyzed.

All parameters have significant differences for different cushions except the contact area. In Ebe and Griffin (2001) and Yongxiang et al. (2019) studies, the contact area also did not have a significant effect. Different shapes of cushions may affect the contact area more directly. This result showed that for cushions with the same configuration and different materials and stiffness, the contact area is not an appropriate parameter to measure comfort. Contact pressure and SPD% are consistent with subjective rating: low maximum pressure and average pressure give people less oppressive feeling, and low SPD% means more uniform pressure distribution, which corresponds to a higher comfort rating. As shown in Figure 8, SPD% has a significant correlation with subjective rating, which proves that among these parameters, it is the most suitable for the evaluation of cushion comfort. Milivojevich et al. (2000) and Oudenhuijzen et al. (2003) also reported that the pressure values, being high or low, do not relate directly to comfort. However, the pressure distribution relates directly to comfort. In addition, the objective parameters are consistent with the subjective rating, and the positive feedback from the subjects proves that the subjective scale and the calculation method used in this study have a good effect on the comfort evaluation.

Compared with the fast-recovery cushion, the contact pressures of the N3 and N4 slow-recovery cushions are obviously lower, the pressure distributions are more uniform, and higher subjective ratings are also obtained. However, due to its higher stiffness, the N2 cushion is the most uncomfortable subjectively and objectively. Jackson et al. (2009) proposed that the peak pressure below 9.3 kPa will not cause evident discomfort within 2 h of riding. The average peak pressure of N2 is 9.0 kPa, which is close to the highest value. A suitable slow-recovery cushion is indeed more comfortable than fast-recovery foam, but the performance of slow-recovery cushions is affected by material property. It is necessary to further determine a comfortable range in order to better exert the ability of the slow-recovery material to evenly distribute the pressure.

As described in Figures 4, 6, comparing the three groups of slow-recovery cushions, we found that the contact pressure and subjective rating change with stiffness are non-linear, which is the same as the result of the study (Ebe and Griffin, 2001). The N3 cushion was rated the most comfortable by both subjective survey and objective measures. The cloud chart can more intuitively see the pressure distribution between different cushions. The participant had the lowest contact pressure while sitting on the N3, and the dispersion was more uniform. The other three cushions can observe obvious pressure peaks at the ischial tuberosity shown in Figure 7. However, the softer N4 cushion is less comfortable. There are two possible reasons: A certain degree of sag will occur when the cushion is under pressure during riding. The softer the cushion with strong deformability, the deeper the sag. The main body thickness of the ejection seat cushion is only 3 cm. After the soft N4 cushion sagging, the compressed bottom layer is not enough to buffer pressure and becomes hard. It makes the value of the contact pressure increase. Kumar et al. (2019) demonstrated that comfort increases with the thickness of seat cushion increase until the thickness reaches 6 cm. The N4 cushion may not be the optimal comfort on a thickness of 3 cm. Another reason is lack of support. Cunningham et al. (1994) believed that a comfortable cushion needs to have both a soft contact surface and deep-down firmness. Wolfe (1982) proposed that a lower 25% IFD can be used to characterize the softness of the contact surface, and a higher 65% IFD represents sufficient support. As shown in Figure 2, with the increase in the deformation, the IFD of N4 did not increase distinctly, and the 65% IFD was the lowest among the four cushions. Insufficient support can also cause cushion discomfort. In short, for the ejection seat cushion, too soft and hard slow-recovery cushions will cause a decline in comfort; the overall performance in comfort of the N3 seat cushion is the best.

For pilots on extended missions, lack of support can affect sitting comfort (Cascioli et al., 2011; Kim et al., 2018). Further analysis of the evaluation of support showed that the support rating was significantly positively correlated with 65% IFD. The result showed that slow-recovery cushions do not have as much support as fast-recovery cushions. The slow-recovery material is temperature-sensitive and becomes soft as the temperature of the contact surface accumulates. As the ride time is extended, its support will slowly decline, and it will be a little muggy as the cushion fitter to the human body. However, the elasticity of fast-recovery material is not affected by temperature and pressure and will maintain constant support.

## Conclusion

In order to study the optimization effect of slow-recovery material on the comfort of the ejection seat cushion, the sitting comfort of 20 participants was tested on four types of cushions by subjective survey and sitting pressure distribution. Subjective ratings were consistent with seat pressure data. In the sitting pressure distribution parameters, SPD% is significantly correlated with the subjective rating, which is more suitable to evaluate the comfort of seat cushions with different properties.

The suitable slow-recovery material could evenly disperse the pressure, and the sitting comfort is significantly better than the fast-recovery seat cushion. Too soft or too hard slow-recovery cushion will lead to a decline in comfort. In the first phase of the study, the N3 seat cushion that has the best sitting comfort on the ejection seat was selected. In other words, when the seat cushion thickness is 3 cm, a slow-recovery cushion with 65% IFD of 280 N is suitable. However, we found that the slow-recovery cushion will show insufficient support and stuffiness after a long ride. And the fast-recovery cushion always maintains a stable state. Therefore, in the next phase of comfort optimization research of ejection seat cushions, we will discuss how to combine the advantages of the two kinds of seat cushions to achieve better results.

The present study only focuses on static comfort, but dynamic comfort is equally important for fighter aircraft. It should be improved in future research. Moreover, in order to better experience the different cushion materials, the experimental cushions were not covered, so thermal comfort was not considered.

## Data Availability

The original contributions presented in the study are included in the article/Supplementary Material; further inquiries can be directed to the corresponding author.
