# Supplementary material for: Comfort Evaluation of Slow-Recovery Ejection Seat Cushions Based on Sitting Pressure Distribution
Source: Front Bioeng Biotechnol. 2021 Nov 30;9:759442. doi: 10.3389/fbioe.2021.759442 (PMC8669618; doi:10.3389/fbioe.2021.759442)
Supplement: Supplementary file 1 [file Presentation1.PDF]

Comfort evaluation of slow-recovery ejection seat cushions based on sitting pressure distribution

frontiers in Bioengineering and Biotechnology

Subject

Participants

The sitting comfort of 10 males and 10 females were test. (n=20)

Ejection Seat Cushions

(Thickness: 3cm)

- N1: fast-recovery foam
- N2: slow-recovery foam (hard)
- N3: slow-recovery foam (mid)
- N4: slow-recovery foam (soft)

Methods

Subjective Survey

7 Comfortable Descriptors

10 Uncomfortable Descriptors

Analytic Hierarchy Process

Subjective Rating

Seat Pressure Distribution

- Max Pressure
- Average Pressure
- Contact Area
- SPD%

Finding

Comfort to Different Materials

Slow-recovery Foam

VS

Fast-recovery Foam

Comfort to Different IFD

The change was nonlinear.

N3 was the best. (65% IFD is 280N)

Correlation with Rating

SPD% is the most suitable for comfort evaluation of different material seat cushions.

(P = 0.019, R = - 0.981)

Further

After a Long Ride

- Fast-recovery cushions maintain a stable state.
- Slow-recovery cushions show insufficient support.

Question in Next Phase

How to combine the advantages of the two kinds of ejection seat cushions?

Reference: Bao, J., Zhou, Q., Wang, X., Yin, C. Comfort evaluation of slow-recovery ejection seat cushions based on sitting pressure distribution. *Frontiers in Bioengineering and Biotechnology*, (2021).
