# Supplementary material for: Comfort Evaluation of Slow-Recovery Ejection Seat Cushions Based on Sitting Pressure Distribution
Source: Front Bioeng Biotechnol. 2021 Nov 30;9:759442. doi: 10.3389/fbioe.2021.759442 (PMC8669618; doi:10.3389/fbioe.2021.759442)
Supplement: Supplementary file 2 [file DataSheet1.PDF]

Table 1 AHP layer A_1 judgment matrix

A_1	Comfort	Discomfort	W_i
**Comfort**	1.00	1.00	0.5
**Discomfort**	1.00	1.00	0.5

Table 2 A_{11} Comfort judgment matrix

A_{11}	relaxed	spirits soared	restful	softer	supported enough	refreshed	comfortable	W_i
relaxed	1.00	1.00	1.00	0.33	0.50	0.50	0.33	0.08
spirits soared	1.00	1.00	1.00	0.33	0.50	0.50	0.33	0.08
restful	1.00	1.00	1.00	0.33	0.50	0.50	0.33	0.08
softer	2.00	2.00	2.00	1.00	1.00	1.00	0.67	0.16
supported enough	2.00	2.00	2.00	1.00	1.00	1.00	0.67	0.16
refreshed	2.00	2.00	2.00	1.00	1.00	1.00	0.67	0.16
comfortable	3.00	3.00	3.00	3.00	3.00	3.00	1.00	0.31

Table 3 A_{12} Discomfort judgment matrix

A_{12}	sore muscles	heavy legs	stiff	tired	swollen ankles	numb	circulation cut off	cramped	restless	uncomfortable	W_i
sore muscles	1.00	1.00	1.00	1.00	1.00	1.00	1.00	1.00	0.50	0.33	0.08
heavy legs	1.00	1.00	1.00	1.00	1.00	1.00	1.00	1.00	0.50	0.33	0.08
stiff	1.00	1.00	1.00	1.00	1.00	1.00	1.00	1.00	0.50	0.33	0.08
tired	1.00	1.00	1.00	1.00	1.00	1.00	1.00	1.00	0.50	0.33	0.08
swollen ankles	1.00	1.00	1.00	1.00	1.00	1.00	1.00	1.00	0.50	0.33	0.08
numb	1.00	1.00	1.00	1.00	1.00	1.00	1.00	1.00	0.50	0.33	0.08
circulation cut off	1.00	1.00	1.00	1.00	1.00	1.00	1.00	1.00	0.50	0.33	0.08
cramped	1.00	1.00	1.00	1.00	1.00	1.00	1.00	1.00	0.50	0.33	0.08
restless	1.00	1.00	1.00	1.00	1.00	1.00	1.00	1.00	1.00	0.33	0.09
uncomfortable	3.00	3.00	3.00	3.00	3.00	3.00	3.00	3.00	3.00	1.00	0.26

Table 4 A_{1j} layer matrix consistency test results

	n	λ_{max}	*C.I.*	*R.I.*	*C.R.*
A_{11}	7	7.27	0.05	1.32	0.03
A_{12}	10	9.58	-0.05	1.49	-0.03

The *C.R.* values of the two judgment matrices are all < 0.1, which have good consistency. The priority scales of each description item are obtained by multiplying the priority W_i of A_{1j} matrix by the priority 0.5 of A_1 layer matrix.
